# Attentional performance is correlated with the local regional efficiency of intrinsic brain networks

**DOI:** 10.3389/fnbeh.2015.00200

**Published:** 2015-07-28

**Authors:** Junhai Xu, Xuntao Yin, Haitao Ge, Yan Han, Zengchang Pang, Yuchun Tang, Baolin Liu, Shuwei Liu

**Affiliations:** ^1^Department of Computer Information and Technology, School of Computer Science and Technology, Tianjin UniversityTianjin, China; ^2^Research Center for Sectional and Imaging Anatomy, Department of Anatomy and Histology Embryology, Shandong University School of MedicineJinan, China; ^3^Department of Radiology, Affiliated Hospital of Medical College, Qingdao UniversityQingdao, China; ^4^Department of Epidemiology, Qingdao Municipal Central for Disease Control and PreventionQingdao, China

**Keywords:** resting state fMRI, functional connectivity, attention, attention network test, graph analysis

## Abstract

Attention is a crucial brain function for human beings. Using neuropsychological paradigms and task-based functional brain imaging, previous studies have indicated that widely distributed brain regions are engaged in three distinct attention subsystems: alerting, orienting and executive control (EC). Here, we explored the potential contribution of spontaneous brain activity to attention by examining whether resting-state activity could account for individual differences of the attentional performance in normal individuals. The resting-state functional images and behavioral data from attention network test (ANT) task were collected in 59 healthy subjects. Graph analysis was conducted to obtain the characteristics of functional brain networks and linear regression analyses were used to explore their relationships with behavioral performances of the three attentional components. We found that there was no significant relationship between the attentional performance and the global measures, while the attentional performance was associated with specific local regional efficiency. These regions related to the scores of alerting, orienting and EC largely overlapped with the regions activated in previous task-related functional imaging studies, and were consistent with the intrinsic dorsal and ventral attention networks (DAN/VAN). In addition, the strong associations between the attentional performance and specific regional efficiency suggested that there was a possible relationship between the DAN/VAN and task performances in the ANT. We concluded that the intrinsic activity of the human brain could reflect the processing efficiency of the attention system. Our findings revealed a robust evidence for the functional significance of the efficiently organized intrinsic brain network for highly productive cognitions and the hypothesized role of the DAN/VAN at rest.

## Introduction

Our brain is a complex network that involves structurally and functionally connected regions. Information is processed and integrated in the brain network with coherent temporal dynamics, and the process is never-ending, even at rest (Sporns et al., 2004; Honey et al., 2009; Van Den Heuvel et al., 2009b). Examining the human brain as an integrative network of functionally interacting regions can bring new insights into the large-scale neuronal communication in the human brain and provide a platform to explore how the functional connectivity and information integration relates to human behavior and cognition and how this architecture may be altered in diseases (Greicius, 2008; Bullmore and Sporns, 2009). Recently, many researchers have made great progresses on exploring the nature of the spontaneous functional activity by examining the slow (< 0.1 Hz) intrinsic blood oxygen level dependent (BOLD) fluctuations in resting state. These studies have suggested that many neuroanatomical systems tend to be highly coherent in the spontaneous activity, including the motor (Biswal et al., 1995; Lowe et al., 1998), auditory (Cordes et al., 2001), visual (Lowe et al., 1998), language (Hampson et al., 2002), default mode (Fox et al., 2005), and attention systems (Fox et al., 2006; Alnaes et al., 2015). Moreover, two segregated attention networks have been identified in the human brain on the basis of spontaneous activity: the dorsal and ventral attention networks (Corbetta and Shulman, 2002; Fox et al., 2006). The first dorsal attention network (DAN) employs the dorsal fronto-parietal areas, including the conjunction of the precentral and superior frontal sulcus (frontal eye field, FEF), medial intraparietal sulcus (IPS), and superior parietal lobule (SPL), dedicating to mediation of goal-directed process and selection for stimuli and responses. The second ventral attention network (VAN), which includes the ventral fronto-parietal areas, including the right temporal parietal junction (TPJ), ventral frontal cortex, and anterior cingulate cortex, is involved in reorienting attention in response to salient sensory stimuli (Fox et al., 2006; Kim, 2014; Farrant and Uddin, 2015). The intrinsic DAN/VAN seems of special relevance for cognitive processes.

Previous studies on the psychological association of the intrinsic brain networks have focused on the activation and deactivation of the brain during task conditions, while only a few have explored the experimental performances on the functional integration in resting state (Fransson, 2005; Mennes et al., 2011). An efficient approach to examining the function of the intrinsic brain networks during rest is to identify the correlations between the intrinsic brain networks and a relevant psychological measure outside the MRI scanning environment. This approach has been applied to investigating several intrinsic brain networks (Seeley et al., 2007; Sheng et al., 2010; Markett et al., 2014), however, the evidence for the DAN/VAN has been scarce. In this study, an attempt was made to seek the relationship between the DAN/VAN and the cognitive capacity in the domain of attention, in the hope to explore the functional role for DAN/VAN in cognitive neuroscience.

Attention refers to both preparedness for and selection of certain aspects of our physical environment (e.g., objects) or some ideas in our mind stored in memory (Raz and Buhle, 2006). Consistent with Posner's earlier accounts of the attention system (Posner and Petersen, 1990), recent functional magnetic resonance imaging (fMRI) studies have supported the notion that there are three key functionally and anatomically distinct subsystems of attention, namely the alerting, orienting, and executive control (EC) systems (Fan et al., 2005; Posner, 2008, 2012, 2014). Specifically, Fan et al. (2002, 2005) have proposed the attention network test (ANT), which provides a means for exploring the behavioral reaction and brain activity of the alerting, orienting, and EC networks in a single integrated task. Since then, ANT has been used in numerous studies with normal populations (Fan et al., 2007a,b; Niogi et al., 2010; Westlye et al., 2011; Joseph et al., 2014) as well as patients with neuropsychiatric disorders, such as attention deficit hyperactivity disorder (ADHD) (Konrad et al., 2006; Adolfsdottir et al., 2008), schizophrenia (Gooding et al., 2006; Urbanek et al., 2009; Backes et al., 2011; Hahn et al., 2011; Diwadkar et al., 2014), autism (Keehn et al., 2010) and multiple sclerosis (Kollndorfer et al., 2013). And ANT task performances outside the MRI environment, have been widely used to explore the relationship between the attentional performances and DAN/VAN in normal subjects (Markett et al., 2014) and patients with neuropsychiatric disorders (Zhang et al., 2015).

Many studies have suggested that there is a direct link between resting-state functional connectivity pattern and human cognitions. These studies have focused on the examination of relationship between the cognitive performances and specific resting-state networks, especially the default mode network (DMN) (Singh and Fawcett, 2008; Van Den Heuvel and Hulshoff Pol, 2010; Deco et al., 2011). Our previous study on attention found that the spontaneous brain activity could predict the attention performance, and the strengths of brain integration (functional connectivity) in specific regions played a quantitatively greater role in predicting the task performance in the ANT (Xu et al., 2014). Another DTI study demonstrated that the behavioral performance of ANT was correlated with the integrity of fiber tracts connecting the key brain regions (Ge et al., 2013). The function of the fronto-parietal network has been assessed by testing the association between intrinsic brain networks and behavioral performances provided by ANT (Markett et al., 2014). The findings that distinct brain regions from neural networks during attention task performances and the fact that some of these regions may contribute to an intrinsic connectivity network suggest a possible relationship between the DAN/VAN and task performances in the ANT.

Although examination of the specific functional connectivity between cortical regions in resting state has been widely used to quantifying the individual differences within an intrinsic brain network, and it could provide new valuable insights into cognition (Van Den Heuvel and Hulshoff Pol, 2010), the application of graph theoretical measures to resting-state signals has been an elegant means to study the relationship between the intrinsic brain networks and task performances (Sporns, 2013a,b). The graph theory analysis has been successfully applied to neuroimaging data by defining regions of interest as nodes and functional connectivity as connecting edges (Van Den Heuvel et al., 2008a; Braun et al., 2012). In the graph theory, the clustering coefficients reflect the information about the local density of information transferring in a network; the characteristic path length provides the information about the level of global communication efficiency of a network. The small-world manner suggests a high level of local neighborhood clustering and a high level of global communication efficiency across the network and integration of information between different regions (Achard et al., 2006; Bullmore and Sporns, 2009; Van Den Heuvel et al., 2009b; Sporns, 2013c). Moreover, the global and local efficiencies can directly estimate the economic performance of small-world brain networks (Achard and Bullmore, 2007). The graph theory analysis provides us a theoretical framework in which the topology of complex networks could be examined, and reveals some important information on both local and global organization of functional brain networks (Stam and Reijneveld, 2007; Bullmore and Sporns, 2009; Stam et al., 2009; Van Den Heuvel and Hulshoff Pol, 2010; Xia and He, 2011; Bullmore and Vertes, 2013). Advanced graph analysis techniques have been applied on resting-state fMRI data in normal subjects (Achard and Bullmore, 2007; Van Den Heuvel et al., 2009a; Davis et al., 2013) as well as patients (Maudoux et al., 2012; Rubinov and Bullmore, 2013; Pironti et al., 2014).

Though the attentional function plays a vital role in cognitive neuroscience, its relationship to the intrinsic human brain integration is still not clear. In this study, we hypothesized a possible relationship between properties of the intrinsic brain networks (DAN/VAN) in the resting brain and behavioral indices of attention capability provided by ANT. To identify how attentional function is maintained at rest in the absence of specific inputs or outputs, the current study constructed intrinsic functional human brain networks at rest using a graph-based approach, and calculated topological properties of the functional network for each subject. Then a linear regression analysis was conducted between the efficiency of the intrinsic brain networks and task performances in the ANT task. Exploring such a relationship would provide more direct evidences that the DAN/VAN at rest played a role in attention, as opposed to the observed activation during task conditions.

## Materials and methods

### Participants

Fifty-nine right-handed healthy subjects (28 females; 17.42 ± 1.42 years old, ranging from 15 to 20 years old) participated in this study, who were recruited from the same sample as our previous study (Xu et al., 2014). Subjects were assessed by a senior neurologist and the Chinese version of the Mini international Neuropsychiatric Interview (Sheehan et al., 1998), which is a standard psychiatric examination validated in the adolescents (Sheehan et al., 2010) and provides diagnosis corresponding to Diagnostic and Statistical Manual of Mental Disorders (DSM-IV) criteria. All the subjects had no history of any neurological or psychiatric disorders, or cognitive complaints, and no abnormal findings was observed in conventional brain MRI. All the subjects were right-handed measured with the Edinburgh handedness inventory (Oldfield, 1971). The study was approved by the Human Research Ethics Committee of Shandong University School of Medicine. Written informed consents were obtained from all the participants, as well as their parents.

### Procedure

A version of ANT devised by Fan and his colleagues (Fan et al., 2005) was adopted to score the behavioral measure after the resting-state fMRI scans. In the ANT task, the participants were instructed to press a button as quickly and accurately as possible to make a decision on the direction of the target (left or right), which was a leftward or rightward arrow at the center. The target was flanked on either side by two arrows; either pointing in the same direction (congruent condition), or in the opposite direction (incongruent condition). The target and flankers were presented until the participant made a response or 2000 ms elapsed. A cue (an asterisk) was presented for 200 ms before the appearance of the target. There were three cue conditions: no cue (baseline), center cue (at the fixation point for alerting) and spatial cue (at the target location for alerting and orienting).

In each block, six trial types (three cue conditions by two target conditions) were presented in a predetermined counterbalanced order. Each subject performed a total of six blocks of trials, with each block lasting 5 min 42 s and consisting of 36 trials plus two buffer trials in the beginning. All the participants were trained before testing. Stimulus presentation and behavioral response collection were performed using E-Prime (Psychology Software Tools, Pittsburgh, PA, USA) on a personal computer outside the scanning room.

### Data acquisition

All the participants were scanned using a 3.0 Tesla GE Signa scanner (General Electric Medical Systems, Milwaukee, WI), with a standard eight-channel head coil. Foam pads and earplugs were used to reduce the head motion and scanner noise. During the resting-state scanning, the subjects were instructed to lie in the scanner, keep their eyes closed, relax their minds and think of nothing in particular without falling asleep. Functional images were obtained using an echo-planar imaging (EPI) sequence: 35 axial slices, thickness/gap = 3.5/0.7 mm, matrix = 64 × 64, repetition time (TR) = 2000 ms, echo time (TE) = 35 ms, flip angle (FA) = 90°, field of view (FOV) = 224 × 224 mm^2^. The resting-state fMRI scan lasted for 5 min 42 s and 171 images were collected for each subject.

In addition, a three-dimensional volume spoiled gradient-echo (SPGR) pulse sequence with 174 slices (TR = 6.5 ms, TE = 2.0 ms, thickness/gap = 1.0/0 mm, matrix = 256 × 256, FOV = 256 × 256 mm^2^, FA = 15°) was used to acquire the structural images for anatomical reference.

### Image preprocessing

Image preprocessing was conducted using SPM8 software (http://www.fil.ion.ucl.ac.uk/spm/). The first 10 volumes for each participant were discarded to allow for T1 equilibration effects and adaptation of participants to the circumstances. The remaining functional images were first corrected for the time delay between slices, and head motion was corrected by a realignment analysis. As all the participants' head movements were less than 1.5 mm and 1 degree, no participant was excluded (translation: 0.91 ± 0.36 mm; rotation: 0.57 ± 0.25°). Next, the realigned images were spatially normalized into a standard stereotaxic space at 3 × 3 × 3 mm^3^, using the Montreal Neurological Institute (MNI) echo-planar imaging (EPI) template. Then, the functional images were spatially smoothed by convolution with an isotropic Gaussian kernel (FWHM = 4 mm) to attenuate spatial noise, and were temporally band-pass filtered (0.01 ~ 0.08 Hz) to reduce the effects of low-frequency drift and high-frequency physiological noises (Biswal et al., 1995). Several sources of spurious variance along with their temporal derivatives were also removed from the data through linear regression: six head motion parameters, averaged signals from cerebrospinal fluid (CSF) and white matter, and global brain signals (Power et al., 2015). This regression procedure removed fluctuations unlikely to be involved in specific regional correlations.

### Behavioral measures

The accuracy of each participant was calculated and those with poor performances (accuracy less than 80%) were excluded in this study (No participant was excluded in this study). Trials with incorrect responses or with response time (RT) longer than 1500 ms or shorter than 200 ms were excluded to avoid possible influences of the outliers. The next response following an error was removed to avoid the post-error slowing effect. Since RTs were not normally distributed, the median RT per condition was used for analysis (Adolfsdottir et al., 2008). The accuracy for each trial type was also calculated. Instead of the conventional subtraction measure (Fan et al., 2005, 2007b), we used ratio scores to define the efficiency of the executive control. The ratio scores, which have been used to explore the structure–behavior correlation (Westlye et al., 2011) and attention impairments (Urbanek et al., 2009), would be more appropriate than RT scores in ANT studies, since the former could isolate the attention system from the overall RT (Ge et al., 2013; Xu et al., 2014). The formulas were as follows:

$$\begin{array}{l} \text{Alerting effect}=\left({\text{RT}}_{\text{no cue}}-{\text{RT}}_{\text{center cue}}\right)∕{\text{RT}}_{\text{center cue}}\\ \text{Orienting effect}=\left({\text{RT}}_{\text{center cue}}-{\text{RT}}_{\text{spatial cue}}\right)∕{\text{RT}}_{\text{spatial cue}}\\ \text{EC effect}=\left({\text{RT}}_{\text{incongruent}}-{\text{RT}}_{\text{congruent}}\right)∕{\text{RT}}_{\text{congruent}} \end{array}$$

### Graph analysis

**Figure 1:**
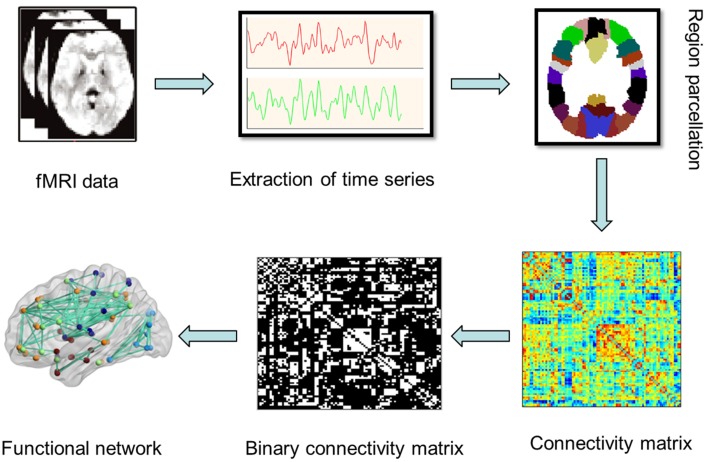
**A flowchar for the construction of functional brain network in resting state fMRI**.

To obtain the large-scale brain network, a prior anatomical automatic labeling (AAL) atlas was used to divide the whole brain into 90 cortical and subcortical regions of interest (45 for each hemisphere). A representative time series was extracted by averaging the time series of all voxels within each region, followed by a Pearson's correlation analysis to calculate the coefficients between each pair of regions, and then Fisher *z* score transformations were conducted for the correlation coefficients to generate a *z*-functional connectivity matrix *A*_*ij*_ = [*a*_*ij*_] (90 × 90) for each subject (Power et al., 2013). Figure 1 showed the flowchar for the construnction of functional brain networks in resting-state fMRI.

An unweighted undirected network was then formed by thresholding the correlation matrix, where the entry *a*_*ij*_ was 1 if the value was larger than the threshold and 0 otherwise. In this study, the threshold range was varied between 0.05 and 0.5, with steps of 0.05. The maximum threshold of 0.5 was defined to minimize possible spurious edges and make sure that all the individual networks have reached 95% full connections (Van Den Heuvel et al., 2008b, 2009b). Next, a number of key topological characteristics that describe the overall architecture of a functional brain network *G* were computed using the Brain Connectivity Toolbox (BCT, http://www.brain-connectivity-toolbox.net/), including the overall clustering coefficient *C*, characteristic shortest path length *L* (Watts and Strogatz, 1998), small-worldness, global efficiency (Latora and Marchiori, 2001) and local nodal efficiency. The definitions and formulas of these graph metrics were listed in the Supplementary Materials.

### Statistical analyses

Statistical analyses were carried out using SPSS 20.0 software. Firstly, independent sample *t*-tests were performed to identify whether there were significant gender differences in the behavioral performances of all the subjects. The correlation analyses were then conducted, with the statistical significance being set at *p* < 0.05. The functional brain networks were constructed based on the correlation threshold *T* = 0.45, and the topological characteristics of the functional brain networks were then calculated. This threshold was determined by satisfying the following criteria. Firstly, the network must differ from a random graph on measure of the small-world properties, indicating the best small-world topology (Bressler and Menon, 2010); secondly, the networks for all the participants were full connected. Then the linear regression analysis was used to explore the association between the behavioral performance of attentional function and brain network organization. To examine the relationship between the organization of the brain networks and attentional function, the topological measures of the individual brain networks, such as small-worldness, shortest path length, and clustering coefficients, were correlated with the behavioral performance of the participants.

To identify the specific brain regions whose local network organization was significantly associated with the behavioral performance of three sub-networks, the correlation analysis was conducted, in which the local efficiency of each individual node was correlated with alerting, orienting and EC effects (Van Den Heuvel et al., 2009b). The individual efficiency of a node reflects how closely this node is connected to the other nodes of the network. First, for each individual dataset, the local efficiency of each node was computed. Second, for all the 90 nodes based on AAL atlas, the efficiency of each node was separately correlated with the measured alerting, orienting and EC effect scores over the whole subjects. This procedure resulted in certain correlated regions indicating which regions showed a significant association with the attentional performance. A false discovery rate (FDR) corrected threshold of 0.05 was considered as significant for the multiple correlation analyses. An exploratory uncorrected analysis was then conducted. The same analysis was made to the mRT scores as we were curious about whether certain regions were in control of people's overall response performance.

## Results

### Behavioral performance

Table 1 showed participants' behavioral performances, including the total mean RT, accuracy and ratio scores. The average accuracy across all the participants was high enough (97%, SD = 0.02), indicating that they followed the instructions and were able to make reliable responses. Independent-sample *t*-tests revealed that there were no significant gender differences for alerting, orienting, EC, mRT, and accuracy.

**Table 1:** **Participants' ratio scores of attention components (Mean ± SD)**.

	**Sample**	**Alerting (%)**	**Orienting (%)**	**EC (%)**	**mRT (ms)**	**Accuracy (%)**
M	31	5.90 ± 3.21	10.57 ± 5.52	16.20 ± 6.27	602.81 ± 60.09	96.68 ± 1.94
F	28	6.11 ± 3.63	9.96 ± 4.19	13.84 ± 4.21	603.26 ± 59.52	97.41 ± 1.87
t (p)		0.24 (0.81)	0.48 (0.63)	1.73 (0.09)	0.03 (0.98)	1.49 (0.14)
Total	59	5.99 ± 3.38	10.30 ± 4.94	15.14 ± 5.53	603.02±59.32	97.01 ± 1.93

*The effects of alerting, orienting and EC are displayed in percent relative to the baseline condition. t, the t-value of independent samples t-test. EC, executive control; RT, response time; M, male; F, female*.

Table 2 summarized the ratio scores of alerting, orienting and EC effects as well as their correlations. Only the correlation between alerting and orienting was significant (*r* = −0.32, *p* = 0.01), after controlling for age and gender. Interestingly, mRT was found to be positively associated with accuracy (*r* = 0.41, *p* = 0.001), and had a negative correlation with age (*r* = −0.35, *p* = 0.006).

**Table 2:** **Correlation between the behavioral performances on attention components**.

	**Alerting**	**Orienting**	**EC**	**mRT**	**Accuracy**
Orienting	−0.32 (0.01^*^)				
EC	0.08 (0.56)	0.11 (0.41)			
mRT	0.12 (0.38)	−0.20 (0.14)	0.04 (0.76)		
Accuracy	−0.11 (0.39)	−0.10 (0.43)	−0.20 (0.12)	0.41 (0.001^**^)	
Age	−0.03 (0.83)	0.25(0.05)	0.16 (0.22)	−0.35 (0.006^**^)	−0.03 (0.80)

** *Correlation is significant at the 0.01 level (2-tailed)*.

* *Correlation is significant at the level 0.05 level (2-tailed). EC, executive control; mRT, median response time*.

### Construction and topology calculation of intrinsic brain networks

**Figure 2:**
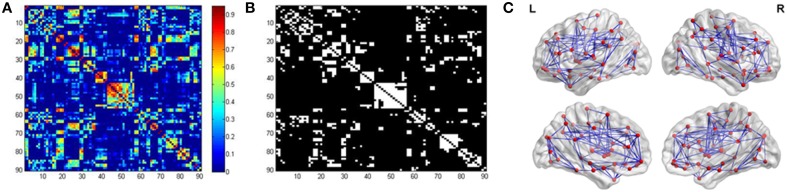
**Inter-regional correlation matrix and functional connectivity backbone**. **(A)** showed the mean correlation obtained by averaging all the correlation matrices across participants. **(B)** showed the binary matrix using threshold *T* = 0.45. **(C)** Visualization of the averaged functional brain network using BrainNet Viewer.

We constructed the functional brain network for each participant, with 90 nodes and 4005 edges [i.e., (90 × 89)/2] by thresholding the correlation matrix. Figure 2 showed the averaged correlation matrix and binary matrix by thresholding *T* = 0.45. The averaged functional brain network was mapped using the BrainNet Viewer software (http://www.nitrc.org/projects/bnv/), as shown in Figure 2.

**Figure 3:**
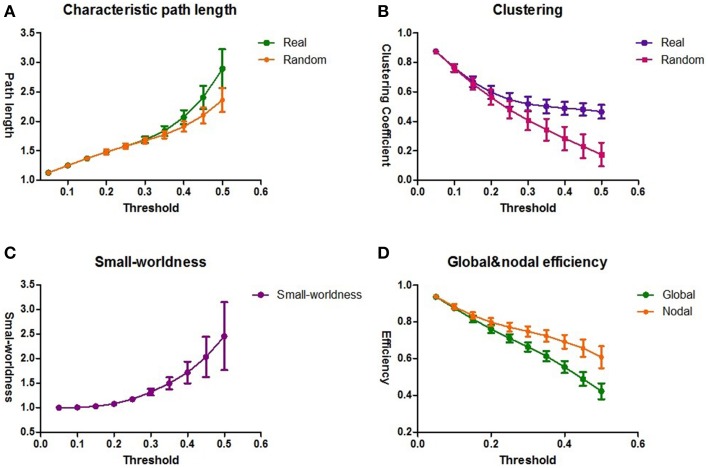
**The topological architecture of functional brain networks**. The results of the group averaged characteristic path length L and L^*rand*^ **(A)** and the clustering coefficient C and C^*rand*^ **(B)** for the thresholds 0.05 ≤ *T* ≤ 0.5. L^*rand*^ and C^*rand*^ reflect the characteristic path length and clustering coefficient of a comparable random network. **(C)** The results of small-worldness for all the functional brain networks. The functional brain networks show a clear small-world organization, expressed by λ≈ 1 and γ >> 1. **(D)** The averaged global and nodal efficiency for all the functional brain networks. The standard deviations are shown as the error bars.

For each threshold (ranging from 0.05 to 0.5), the graph characteristic measures were averaged over the group of 59 subjects. The intrinsic brain networks showed a clear small world architecture for 0.35 ≤ *T* ≤ 0.5 (Figure 3), expressed by L ≈ L^*rand*^, and λ≈ 1 for *T* ≤ 0.5 and C >> C^*rand*^ and γ >> 1 for *T* ≥ 0.35 (one-sample *t*-test for λ and γ, two-sample *t*-test for L and C; *p* = 0.05, *df* = 58, Bonferroni corrected for multiple comparisons of *T*) (Sporns et al., 2004; Achard et al., 2006; Van Den Heuvel et al., 2009b).

### Correlation between attention behavioral performances and intrinsic brain organization

The association between brain network characteristics and individual behavioral variation in attentional function was examined by correlating mRT score with the lambda λ and gamma γ values of the individual brain networks within the clear small-world range of 0.35 = *T* = 0.5 (Van Den Heuvel et al., 2009b). For the entire threshold, no significant correlation was found between the characteristic path length λ and mRT, and no pronounced distinction existed during the range of the threshold. In addition, the association between mRT and the clustering coefficient γ was also not significant. The same analysis was applied to the sub-networks. Again, there was no significant correlation between the sub-networks and the global brain intrinsic characteristics λ and γ.

### Regions associated with the alerting, orienting and executive control

**Figure 4:**
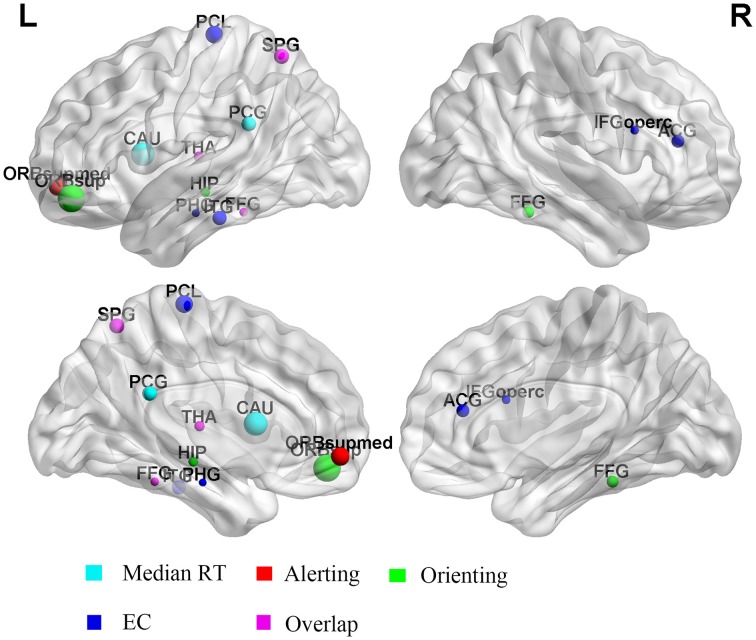
**Regions whose local efficiency was correlated with alerting, orienting, and EC effects**. The radius of the dot represents the correlation coefficient. The color of the dot represents the different components. L, left; R, right.

To further explore whether the performance of attentional function was correlated with the regional brain characteristics, and which specific brain regions had a strong association between the brain network organization and behavioral performances, a linear regression analysis (*p* < 0.05, corrected for age) was conducted between the mRT, alerting, orienting, and EC scores and the local efficiency of each individual node at the threshold *T* = 0.45. After the FDR correction, only one region (left superior frontal gyrus) was discovered whose local efficiency was significantly correlated with the orienting effect. For alerting and EC effects, no significant correlations were found. So an uncorrected exploratory analysis was then conducted. More regions were discovered, and most of the regions were derived. Figure 4 showed the regions that were correlated with each component in the uncorrected analysis.

The uncorrected analysis revealed that the prominent effects between the mRT and the local efficiency were located in the left posterior cingulate gyrus and left caudate nucleus. The left superior parietal gyrus and thalamus were found to be correlated with the alerting effect. The strongest positive effects between the local efficiency and orienting were in the frontal area (left superior frontal gyrus: *r* = 0.421, *p* < 0.001), and some other regions, including the right fusiform gyrus, left fusiform and left superior parietal gyrus. For the EC effect, the right inferior frontal gyrus and anterior cingulate gyrus, left fusiform gyrus and thalamus were discovered. Table 3 summarized the details of regions whose local efficiency was correlated with the alerting, orienting, and EC effects.

**Table 3:** **Regions associated with three components of attention (***p*** < 0.05, uncorrected)**.

**Regions**	***r (p)***
**mRT**	
L Caudate Nucleus	0.344 (0.003)
L Posterior cingulate gyrus	0.279 (0.015)
**ORIENTING**	
L Superior frontal gyrus^b^	0.421 (< 0.001)
R Fusiform gyrus	0.314 (0.008)
L Fusiform gyrus	0.281 (0.014)
L Superior parietal gyrus	−0.26 (0.022)
L Hippocampus	0.254 (0.023)
**ALERTING**	
L Superior frontal gyrus^a^	0.315(0.007)
L Superior parietal gyrus	−0.315 (0.007)
L Thalamus	0.305 (0.008)
**EXECUTIVE CONTROL**	
L Inferior temporal gyrus	−0.364 (0.002)
L Paracentral lobule	0.311(0.007)
R Inferior frontal gyrus^c^	−0.299 (0.01)
R Anterior cingulate gyrus	−0.294 (0.011)
L ParaHippocampal gyrus	−0.269 (0.017)
L Thalamus	−0.255 (0.024)
L Fusiform gyrus	0.254 (0.023)

a *, medial orbital part of superior frontal gyrus*;

b *, orbital part of superior frontal gyrus*;

c *, opercular part of inferior frontal gyrus*.

*L, left; R, right*.

## Discussion

In this study, we intended to delineate the relationship between the attention behavioral performance and the characteristics of the functional brain networks during resting state. The main finding of this study was that the ANT performance showed a strong association with the level of local efficiency, but not with the global communication efficiency. The linear regression analysis revealed that the regions that were correlated with the alerting, orienting and EC effects largely overlapped with the intrinsic DAN/VAN, which was one of the major intrinsic functional networks at rest (Fox et al., 2006). This finding suggested that the intrinsic functional networks at rest could be reflected in the task performance outside the MRI environment, which might make a great contribution to understanding the neuronal mechanism of attention.

We found that the behavioral scores of ANT had no correlation with the global brain characteristics, such as λ and γ. However, a number of studies have suggested that the cognitive performance is correlated with the global efficiency of the intrinsic brain network. A recent study on working memory found that the intrinsic resting-state activity could predict not only the brain activation during working memory but also the behavioral performance (Zou et al., 2013). Another EEG study investigated the relationship between psychometric intelligence and brain functional networks using intracortical current densities and graph-theoretical analysis, and it showed that psychometric intelligence associated with small-world network properties of functional brain networks in resting state (Langer et al., 2012). Specially, we found that the behavioral performance was correlated with the regional efficiency of the brain networks, and the related regions seemed to be integrated into the intrinsic brain networks: DAN/VAN. This suggested that there was a possible relationship between several specific regions of the intrinsic DAN/VAN and task performances in the ANT. We speculated that the human brain was evolved to be more complex and efficient, which could process the external information in an explicit way.

### Correlation between the attentional performance and the global properties of the functional brain networks

The graph analysis of resting state fMRI data has suggested that the human brain is organized to be a cost-effective and efficient small-world topology with the optimization toward a high level of information integration and processing across distinct subnetworks in the human brain (Van Den Heuvel and Hulshoff Pol, 2010). The resting-state functional brain networks constructed in this study demonstrated the small-world topological architecture. And the intra-relationship between the intrinsic low-frequency fluctuation and cognitive behavior has been a new focus on cognitive neuroscience in recent years. And one resting-state study in our group suggested that the regional fluctuations and strengths of functional connectivity in resting state were associated with the behavioral performance in executive control of attention (Xu et al., 2014). However, these studies focused on the relationship between functional connectivity and behavioral performance, while the functional network patterns could act as a powerful predictor for cognitive ability. Using graph analysis, recent neuroimaging studies have explored the relationships between the architecture of functional brain networks and cognitive performance, such as intellectual performance (Song et al., 2008; Van Den Heuvel et al., 2009b; Langer et al., 2012), working memory (Sala-Llonch et al., 2012; Zou et al., 2013), and conceptual processing (Wei et al., 2012). One previous EEG study found that the low performance in intelligence tests was related to changes in the characteristics of the small-world networks that reflected a less optimal topological organization (Langer et al., 2012). In the current study, the relationship between the attentional behavioral performance and the global characteristics of resting-state functional brain networks was explored using the linear regression analysis. Unfortunately, no significant correlations were discovered, which was inconsistent with previous studies. We speculated that the attention behavior performance might have a great association with the local organization of the resting-state functional brain networks. Therefore, our finding was inspiring, which could provide us a better understanding in the neural origin of attention.

### Correlation between the attentional behavioral performance and the local regional efficiency of brain networks

The main purpose of this study was to identify the relationship between the intrinsic functional brain architecture (DAN/VAN) and the attention cognitive performance. The attentional behavioral performance had no correlation with the global brain characteristics, but it was associated with the local efficiency of several specific regions. It is notable that strong positive correlations were found between the attention behavior and all the ANT-activated regions, which suggested that the intrinsic human brain activity could predict the human behavioral performance.

The most prominent effects for the alerting and orienting performances were found in the left superior frontal gyrus (medial orbital part for alerting, and orbital part for orienting), which was close to the FEF. The FEF has been suggested to play a crucial role in the DAN, concerning with orienting ones focus on a particular task (Corbetta et al., 2008). The DAN exerts sustained activation when focusing attention on an object, and it is thought to be responsible for goal-directed, top-down processing (Corbetta and Shulman, 2002). In this study, we also discovered the SPL and fusiform gyrus (part of the motion-sensitive middle temporal area) in the correlation analysis for the alerting and orienting effects, which was involved in the intrinsic DAN. The inferior temporal gyrus and inferior frontal gyrus (opercular part) were significantly correlated with the EC effects, which are the key nodes of the VAN (Corbetta and Shulman, 2002). The VAN reflects the detection of salient stimuli and is generally activated when an unexpected event occurs and breaks one attention from the current task (Corbetta et al., 2008; Kim, 2014), which fits with the functional role of the EC network. The key function of the VAN is to direct attention to stimuli outside of the current focus, and is referred to as the circuit breaking section of the two attention networks (Shulman et al., 2002; Farrant and Uddin, 2015). The anterior cingulate cortex (ACC) was also discovered, which was involved in a domain process of monitoring conflict in the EC of attention (Fan et al., 2005), and the ACC played a functional role in the VAN. Our findings confirmed the relationship between the DAN/VAN and subnetworks of attention (alerting, orienting, and EC). An ANT study also suggested that the topological in frontal-parietal attentional network had a statistical correlation with the behavioral performance (Markett et al., 2014). Our previous DTI study using ANT also demonstrated that the behavioral performance of attention subnetworks had relationships with the quality of white matter tracts (Ge et al., 2013). The current study demonstrated that the alerting, orienting, and EC attention detected by ANT task could reflect the local pattern of several specific regions in the intrinsic brain networks. Previous studieshave suggested that the dorsal/ventral attention model was presented in the ongoing intrinsic brain activity (Sporns et al., 2004; Fox et al., 2006). Our results further reinforced the notion that the task-dependent cognition could be reflected in spontaneous brain fluctuations. Taken together, we suggested that the intrinsic brain network could be reflected in the task performance, and there was a relationship between the specific regions of the intrinsic DAN/VAN and attentional performance assessed by ANT.

### Limitations

There are some limitations to be announced in this study. (1) Parcellation scheme: The parcellation scheme can impress a great impact on the definition of the brain network, which is an important issue in a graph analysis (Fornito et al., 2010). In our study, we divided the human brain into 90 regions based on the AAL-atlas, an effective parcellation scheme at the macroscale. Compared with the region-based networks (e.g., AAL), the voxel-based scheme is an alternative choice whose networks exhibit more desirable properties but less integrate information in the larger brain regions (Hayasaka and Laurienti, 2010). (2) Choice of the threshold: After the functional connectivity between each two regions was calculated, a threshold was set to construct the functional brain networks. Thus, the threshold could have a great influence in the architecture of the functional brain network. We varied the threshold from 0.05 to 0.5, with steps of 0.05. A correlation analysis was made between the attention behavioral performance and the global measures of the brain network for 0.35 ≤ *T* ≤ 0.5. To identify the regions related to the attention network, the threshold of *T* = 0.45 was chosen, because the networks for all the participants were fully connected and represented the best small-world topology (Stevens et al., 2012). (3) Age range of the subjects: the population in this study falls within a narrow age range and is in a developing stage. Our findings may vary with time and be inconsistent with findings in studies on children or adults. A further study needs to be conducted in a wider age range. (4) Multiple comparison problem: The FDR was applied to do the corrections for the multiple comparisons in this study. After the FDR correction, only one region was found to be significant. Then we used an exploratory analysis which was the same as a previous study (Van Den Heuvel et al., 2009b). Some interesting results were discovered, in which some regions were involved in the dorsal and ventral attention network. We speculate that these findings could provide new evidences to understanding the neural origin of attention. Although the exploratory analysis used in this study is a limitation, most of the results are derived.

## Conclusions

In this study, we constructed the intrinsic functional brain networks and identified the small-worldness of the functional brain networks. The linear regression analysis suggested that the attention performance was not associated with the global communication efficiency, but correlated with the local regional efficiency. Our findings confirmed the relationship between the intrinsic DAN/VAN and the task performance in the ANT. Our findings can provide a new insight toward understanding the neural origin of attentional function and open a new window into exploring the spontaneous neuronal activity of the human brain. And our findings suggested that the topology-based approach in resting state could provide an efficient means to reveal the potentially biological mechanism that could be responsible for brain dynamics and the underlying pathophysiology in brain diseases.

### Conflict of interest statement

The authors declare that the research was conducted in the absence of any commercial or financial relationships that could be construed as a potential conflict of interest.
