# Supplementary material for: Attentional performance is correlated with the local regional efficiency of intrinsic brain networks
Source: Front Behav Neurosci. 2015 Jul 28;9:200. doi: 10.3389/fnbeh.2015.00200 (PMC4517058; doi:10.3389/fnbeh.2015.00200)
Supplement: Supplementary file 1 [file DataSheet1.DOCX]

***Supplementary Material***

**Attentional Performance is Correlated with the Local Regional Efficiency of Intrinsic Brain Networks**

**Junhai Xu^1,2^, Xuntao Yin^2^, Haitao Ge^2^, Yan Han^3^ , Zengchang Pang^4^, Yuchun Tang^2^, Baolin Liu^1*^, Shuwei Liu^2*^**

^1^School of Computer Science and Technology, Tianjin University, Tianjin 300072, P.R.China

^2^Research Center for Sectional and Imaging Anatomy, Shandong University School of Medicine, Jinan, Shandong, China

^3^Department of Radiology, Affiliated Hospital of Medical College, Qingdao University, Qingdao, Shandong, China

^4^Department of Epidemiology, Qingdao Municipal Central for Disease Control and Prevention, Qingdao, Shandong, China

*** Correspondence:** Prof. Shuwei Liu, Research Center for Sectional and Imaging Anatomy, Shandong University School of Medicine, Jinan, Shandong, 250012, China. Telephone: +86-531-88382093. Fax: +86-531-88563495.

E-mail address: [liusw@sdu.edu.cn](mailto:liusw@sdu.edu.cn)

Prof. Baolin Liu, School of Computer Science and Technology, Tianjin University, Tianjin, 300072, China. Telephone: +86-22-27406082. Fax: +86-22-27406082.

E-mail address: liubaolin@tsinghua.edu.cn

1. **Supplementary Data**

An N×N binary graph, G, consisting of nodes (brain regions) and undirected edges (functional connectivity) between nodes. We define the subgraph $G_{i}$ as the set of nodes that are the direct neighbors of the $i^{_{th}}$node, i.e., directly connected to the i^th^ node with an edge. Here we just provide a short introduction to the metrics used in this study.

**Degree** K: The degree of each node,$K_{i, i=1,2,...,\text{N}}$, is defined as the number of nodes in the subgraph$G_{i}$. The degree of connectivity, $K_{p}$, of a graph is the average of the degrees of all the nodes in the graph:

$K_{p}=\frac{1}{N}\sum_{i\in G} K_{i}$,

which is a measure to evaluate the degree of sparsity of a network.

**Characteristic shortest path length**$L$: $L_{i}$describes the average number of connections that travel from node $i$ to every other node in the network.

$L_{i}=\frac{1}{N-1}\sum_{i\neq j\in G} \min\{L_{i,j}\}$,

in which $\min\{L_{i,j}\}$is the shortest absolute path length between the $i^{_{th}}$node and the $j^{_{th}}$node, and the shortest absolute path length is the number of edges along the path connecting two nodes. The characteristic shortest path length of a network $L$is the average of the shortest absolute path lengths between the nodes, which is a basis for measuring integration of a network.

$L=\frac{1}{N}\sum_{i\in G} L_{i}$,

$L$ is a measure of the extent of global efficiency or the ability for information propagation in parallel of the network.

**Overall clustering coefficients** $C$: The clustering coefficient of a node $i$ is defined as the ratio of the number of existing connections to the number of all possible connections in the sub-graph$G_{i}$:

$C_{i}=\frac{E_{i}}{K_{i}(K_{i}-1)/2}$,

where $E_{i}$ is the number of edges in the sub-graph $G_{i}$. The overall clustering coefficient of a network is the average of the clustering coefficients of all nodes:

$C=\frac{1}{N}\sum_{i\in G} C_{i}$,

$C$ is a measure of the extent of the local density or cliquishness of information transfer on the network.

**Small-worldness**: Compared with random networks, small-world networks have similar characteristic shortest path lengths but higher overall clustering coefficients ([Watts and Strogatz, 1998](#_ENREF_69)), that is

$$\gamma=C_{p}^{real}/C_{p}^{rand}>1, \lambda=\text{L}_{p}^{real}/\text{L}_{p}^{rand}\approx1$$

These two conditions can be summarized into a scalar quantitative measurement,$\sigma=\gamma/\lambda$, which is typically greater than 1 for small world networks ([Achard et al., 2006](#_ENREF_2); [Humphries et al., 2006](#_ENREF_32); [He et al., 2007](#_ENREF_29)). In details, the values of $C_{p}^{real}$and $L_{p}^{real}$of the functional brain network are compared with those of random networks. The theoretical values of these two measures for random networks are $C_{p}^{rand}=K/N,$ and $L_{p}^{rand}\approx\ln(N)/\ln(K)$([Achard, et al., 2006](#_ENREF_2); [Bassett and Bullmore, 2006](#_ENREF_3); [Stam, et al., 2007](#_ENREF_24)). To obtain a better control for the functional brain networks, here we generated 100 random networks for each degree K by a Markov-chain algorithm ([Maslov and Sneppen, 2002](#_ENREF_17); [Milo, et al., 2002](#_ENREF_19); [Sporns and Zwi, 2004](#_ENREF_23)). In the original matrix, if i_1_ was connected to j_1_ and i_2_ was connected to j_2_, for random matrices, we removed the edge between i_1_ and j_1_ but added an edge between i_1_ and j_2_. That means that a pair of vertices (i_1_,j_1_) and (i_2_,j_2_) was selected for which $\text{e}_{\text{i}_{1}\text{j}_{1}}\text{ }=\text{ 1}$, $\text{e}_{\text{i}_{2}\text{j}_{2}}\text{ }=\text{ 1}$, $\text{e}_{\text{i}_{1}\text{j}_{2}}\text{ }=\text{ 0}$, and $\text{e}_{\text{i}_{2}\text{j}_{1}}\text{ }=\text{ 0}$. Then$\text{e}_{\text{i}_{1}\text{j}_{1}}\text{ }=\text{ 0}$,$\text{e}_{\text{i}_{2}\text{j}_{2}}\text{ }=\text{ 0}$, $\text{e}_{\text{i}_{1}\text{j}_{2}}\text{ }=\text{ 1}$ and $\text{e}_{\text{i}_{2}\text{j}_{1}}\text{ }=\text{ 1}$. Then we randomly permuted the matrices which assured that the random matrix had the same degree distribution as the original matrix. This procedure was repeated until the topological structure of the original matrix was randomized ([Achard, et al., 2006](#_ENREF_2)). Then we averaged across all 100 generated random networks to obtain a mean $C_{p}^{rand}$and a mean $L_{p}^{rand}$ for each degree K.

**Global efficiency：**The global efficiency of a node is:

The global efficiency of the network is:

which is the inverse of the harmonic mean of the shortest path lengths of each pair of nodes, demonstrating the global efficiency of parallel information transfer in the network ([Latora and Marchiori, 2001](#_ENREF_15)).

**Local efficiency**: The local efficiency of a node $i$ is:

which indicates how efficient the information transfers in $G_{i}$ when the node $i$ is eliminated ([Achard and Bullmore, 2007](#_ENREF_1)).

The local efficiency of the network is:

which is the mean local efficiency across all nodes in the network.

References

Achard, S., and Bullmore, E. (2007). Efficiency and cost of economical brain functional networks. *PLoS Comput Biol* 3**,** e17.

Achard, S., Salvador, R., Whitcher, B., Suckling, J., and Bullmore, E. (2006). A resilient, low-frequency, small-world human brain functional network with highly connected association cortical hubs. *J Neurosci* 26**,** 63-72.

Bassett, D.S., Bullmore, E. (2006) Small-world brain networks. Neuroscientist, 12:512-23.

He, Y., Chen, Z.J., and Evans, A.C. (2007). Small-world anatomical networks in the human brain revealed by cortical thickness from MRI. *Cereb Cortex* 17**,** 2407-2419.

Humphries, M.D., Gurney, K., and Prescott, T.J. (2006). The brainstem reticular formation is a small-world, not scale-free, network. *Proc Biol Sci* 273**,** 503-511.

Latora, V., Marchiori, M. (2001) Efficient behavior of small-world networks. Physical review letters, 87:198701.

Maslov, S., Sneppen, K. (2002) Specificity and stability in topology of protein networks. Science, 296:910-3.

Milo, R., Shen-Orr, S., Itzkovitz, S., Kashtan, N., Chklovskii, D., Alon, U. (2002) Network motifs: simple building blocks of complex networks. Science, 298:824-7.

Sporns, O., Zwi, J.D. (2004) The small world of the cerebral cortex. Neuroinformatics, 2:145-62.

Stam, C.J., Jones, B.F., Nolte, G., Breakspear, M., Scheltens, P. (2007) Small-world networks and functional connectivity in Alzheimer's disease. Cereb Cortex, 17:92-9.

Watts, D.J., Strogatz, S.H. (1998) Collective dynamics of 'small-world' networks. Nature, 393:440-2.
